# Antimicrobial photodynamic therapy—a promising treatment for prosthetic joint infections

**DOI:** 10.1007/s10103-017-2394-4

**Published:** 2017-12-15

**Authors:** Timothy Briggs, Gordon Blunn, Simon Hislop, Rita Ramalhete, Caroline Bagley, David McKenna, Melanie Coathup

**Affiliations:** 1 Royal National Orthopaedic Hospital, HA7 4LP, Brockley Hill, HA7 4LP Stanmore UK; 2 Institute of Orthopaedics and Musculo-Skeletal Science, Division of Surgery & Interventional Science, University College London, Royal National Orthopaedic Hospital, Brockley Hill, Stanmore, Middlesex, HA7 4LP UK

**Keywords:** Photodynamic therapy, Methylene blue, Prosthetic joint infection, Biofilms

## Abstract

Periprosthetic joint infection (PJI) is associated with high patient morbidity and a large financial cost. This study investigated Photodynamic Therapy (PDT) as a means of eradicating bacteria that cause PJI, using a laser with a 665-nm wavelength and methylene blue (MB) as the photosensitizer. The effectiveness of MB concentration on the growth inhibition of methicillin-sensitive *Staphylococcus aureus* (MSSA), methicillin-resistant *Staphylococcus aureus* (MRSA), *Staphylococcus epidermidis*, *Pseudomonas aeruginosa* and *Acinetobacter baumannii* was investigated. The effect of laser dose was also investigated and the optimized PDT method was used to investigate its bactericidal effect on species within planktonic culture and following the formation of a biofilm on polished titanium and hydroxyapatite coated titanium discs. Results showed that Staphylococci were eradicated at the lowest concentration of 0.1 mM methylene blue (MB). With *P. aeruginosa* and *A. baumannii*, increasing the MB concentration improved the bactericidal effect. When the laser dose was increased, results showed that the higher the power of the laser the more bacteria were eradicated with a laser power ≥ 35 J/cm^2^ and an irradiance of 35 mW/cm^2^, eradicating all *S. epidermidis*. The optimized PDT method had a significant bactericidal effect against planktonic MRSA and *S. epidermidis* compared to MB alone, laser alone, or control (no treatment). When biofilms were formed, PDT treatment had a significantly higher bactericidal effect than MB alone and laser alone for all species of bacteria investigated on the polished disc surfaces. *P. aeruginosa* grown in a biofilm was shown to be less sensitive to PDT when compared to Staphylococci, and a HA-coated surface reduced the effectiveness of PDT. This study demonstrated that PDT is effective for killing bacteria that cause PJI.

## Introduction

Although the incidence of periprosthetic joint infection (PJI) is low, it results in substantial morbidity and is a considerable financial burden to the healthcare system. Whilst antibiotics are the traditional method used to treat implant infection, these have increasingly limited efficacy. Photodynamic Therapy (PDT) has the ability to kill various microorganisms when the appropriate photosensitizer and light are combined in the presence of oxygen [1].

In primary hip and knee replacement surgery, the rate for deep infection is reported to vary between 0.28 to 4% and 0.39 to 3.9%, respectively, with a higher incidence of infection seen in spinal implants [2–5]. In 2012, the National Joint Registry (NJR) for England and Wales reported a 12% revision rate in total hip replacement arthroplasty (THA) with 12% of those revised due to infection [6]. The lifetime risk of PJI from a haematogenous cause following Staphylococci bacteraemia is 34% [7], and this can increase sixfold in patients with high-risk factors such as obesity, diabetes mellitus, malignancy, smoking and alcohol abuse [8–11]. Revision surgery due to infection is challenging and in the USA; it is projected to exceed US$1.62 billion by 2020 [12]. The mean operative time, the estimated blood loss and complication rate are all higher, and these complications are associated with extended care and increased mean hospital stay, which are estimated to add a 3.6-fold increase to costs [13].

A bacterial biofilm has been defined as “a structured community of bacterial cells enclosed in self-produced polymeric matrix and adherent to an inert or living surface” [14]. PJI is associated with biofilm formation on the implant surface where the bacteria become resistant to the immune system and to antibiotic therapy, and are therefore very difficult to eradicate. PJIs caused by bacterial biofilms are a serious complication of arthroplasty and implant fixation. At present, the most common treatment involves two-stage surgery with removal of the implant, long-term antimicrobial therapy and eventual re-implantation. Despite many novel therapies that try to prevent or disrupt biofilms, there is presently no effective treatment specifically for PJI.

Effective antimicrobial PDT requires use of a photosensitizer and a light source of the appropriate wavelength in the presence of oxygen to form a free-radical ‘singlet oxygen’ that is cytotoxic to targeted microorganisms [15]. Exposure time influences the amount of singlet oxygen formed, causing oxidative damage to prokaryotic and eukaryotic cells by targeting sub-cellular components (e.g. mitochondria, nuclear envelope or cytoplasmic membrane) [16, 17]. This results in cellular disruption producing sterility by a process of necrosis [18]. The power of light and the time of exposure are crucial to successful treatment. A weak light dose will not form enough singlet oxygen molecules, whilst too high a dose damages surrounding healthy eukaryotic tissues. An ideal photosensitizer has strong absorbance of light, excellent photochemical reactivity, minimal dark toxicity (i.e. it is only cytotoxic in presence of light) and is chemically pure. Methylene blue (MB) has not only been shown to be bactericidal when used in PDT, but also preserves the viability and function of host neutrophils [19, 20]. MB has been successfully used to treat periodontal infections in dentistry [21].

Several in vitro studies have investigated the use of antimicrobial PDT and results are promising. Studies using various photosensitizers have demonstrated a complete bactericidal effect on a number of Gram-positive bacteria [22]. However, there appears to be a difference in susceptibility to PDT between Gram-positive and Gram-negative bacteria. Gram-negative bacteria such as *Pseudomonas aeruginosa* are much harder to treat due to different cytoplasmic membrane structures within the bacterial cell wall [23].

PDT can target the area requiring treatment with few systemic side-effects; it is quick, can effectively target bacteria over surrounding tissue and is relatively inexpensive. The antimicrobial effect of PDT on biofilms have been extensively studied in dental plaques and are very promising but few studies have looked at the effect of PDT on infected joint prostheses. If PDT can be developed as a treatment for PJIs, either alone or synergistically with other antimicrobial therapies, then it has the potential to transform orthopaedic practice and significantly lower morbidity and mortality. Our hypothesis was that photodynamic therapy is an effective means of eradicating common strains of bacteria that cause periprosthetic joint infection within planktonic culture, and in a biofilm culture on both polished titanium surfaces and HA-coated implant surfaces.

## Materials and methods

Methicillin-sensitive *Staphylococcus aureus* (MSSA), methicillin-resistant *Staphylococcus aureus* (MRSA), *Staphylococcus epidermidis*, *Pseudomonas aeruginosa* and *Acinetobacter baumannii* were sourced from the bio-resource centers NCTC (UK National Collection of Type Cultures) and ATCC (American Type Culture Collection). *P. aeruginosa* was grown on MacConkey Agar Salt (Oxoid Limited, Thermo Fisher Scientific, Perth, UK) whilst the other species were grown on Columbia agar with 5% horse blood (Oxoid Limited, Thermo Fisher Scientific, Perth, UK). Plates were incubated for 24 h at 37 °C.

### Optimal methylene blue concentration

In order to determine the most effective concentration of MB, all five species were cultured as a lawn onto agar and exposed to increasing concentrations of MB. A visible laser light of 665-nm wavelength (Modulight, Tampere, Finland) was used to activate the photosensitizer and plates were exposed to a laser dose of 35 J/cm^2^ and an irradiance of 35 mW/cm^2^ for 16 min. Each agar plate was marked with six divisions and a 20-μl aliquot of MB at a concentration of 0.1, 0.2, 0.3, 0.4 and 0.5 mM, was pipetted on the agar surface and allowed to spread out. A control group of MB alone was investigated and two repeats per group were carried out. Plates were incubated at 37 °C for 24 h and underwent qualitative visual analysis to assess the bactericidal effectiveness of the different MB concentrations investigated.

### Optimal laser dose

To determine the most effective laser dose required, a bacterial lawn of *S. epidermidis* was cultured onto agar. *S. epidermidis* was chosen due to its higher resistance to PDT (as noted during the previous experiment) and was cultured using the same technique as described above. A 20-μl aliquot of 0.3 mM MB was dropped onto each agar plate and allowed to spread out. Each plate was then exposed to different doses of laser (15, 25, 30, 35, 40 and 45 J/cm^2^) for 16 min, and a control was performed where plates were not exposed to laser. Plates were incubated at 37 °C for 24 h and underwent qualitative visual analysis to assess the bactericidal effectiveness of the different laser doses. Two repeats per experiment were carried out.

### Effect of PDT on planktonic culture

The effectiveness of PDT in eradicating planktonic bacteria was investigated using MRSA and *S. epidermidis* by exposing each culture to four treatments: PDT (MB + L+), photosensitizer alone (MB+L−), laser alone (MB−L+), and control (MB−L−). Each specie was cultured in a suspension in order to achieve the equivalent to a 1 McFarland standard. In the first group, 0.6 mM of MB was added to each suspension and plates were incubated for 5 min at room temperature in the dark to allow bacterial cells to absorb the photosensitizer. Wells were then exposed to a dose of 35 J/cm^2^ and an irradiance of 35 mW/cm^2^ for 16 min. In the second group, MB was added to each of the wells but not exposed to laser light. The third group was treated with laser only, i.e. 35 J/cm^2^ laser light was shone on the well plates with no added MB. The fourth group was used as a control and received no further treatment. For each species, four repeats were performed. Following treatment, the number of remaining bacteria was quantified using a standard serial dilution technique.

### Effect of PDT on biofilm

The effectiveness of PDT in eradicating bacteria in a biofilm was investigated by growing bacteria on both polished titanium alloy and hydroxyapatite (HA)-coated discs. Surgical grade titanium alloy (Ti-6Al-4 V) discs 13 mm diameter × 3 mm thick remained either uncoated (with a polished surface) or were coated with a highly crystalline (> 85%) 50-μm thick plasma sprayed HA coating (Accentus Medical® (Harwell Oxfordshire). Four species were tested (MSSA, MRSA, *S. epidermidis*, and *P. aeruginosa*) on the polished surface and only *P. aeruginosa* was investigated in the HA-coated disc group. In both the coated and uncoated groups, four treatment regimes were applied: PDT (MB+L+), photosensitizer alone (MB+L−), laser alone (MB−L+) and control (MB−L). Bacterial suspensions equivalent to 1 McFarland standard were cultured in Nutrient broth (Oxoid Limited, Thermo Fisher Scientific, Perth, UK) and a serial dilution of each suspension was performed to estimate the number of colony-forming units per milliliter (CFU/ml). For all experiments a total of six repeats was performed for each specie and treatment with and without a HA coating.

Sterilized polished titanium alloy and HA-coated discs were placed within wells in a 24-well plate, and flooded with 1 ml of each bacterial suspension ensuring the surface was covered. To form a biofilm, the well plate was shaken at 80 rpm and incubated at 37 °C for 3 days. The media was removed and each disc was washed gently with phosphate buffered saline (PBS) to remove planktonic bacteria, leaving only the adherent bacteria on the surface of the disc.

For those discs treated with PDT (MB+L+), 0.75 ml of 0.3 mM MB was added to each well and plates were incubated for 5 min at room temperature in the dark. Wells were then exposed to a laser light dose of 35 J/cm^2^ and irradiance of 35 mW/cm^2^ for 16 min. In the second group, MB was added but wells were not exposed to laser light. The third group was treated with laser only, i.e. laser light was shone on the well plates with no added MB. Discs in the control group had no further treatment applied.

Following treatment, discs were washed gently with PBS and sonicated for 10 min at 50–60 Hz to remove adherent bacteria. The suspension was used in a standard serial dilution technique and the number of CFU/ml was quantified.

### Statistical analysis

Data obtained was analyzed using IBM SPSS Statistics (Version 21). A Kolmogorov–Smirnov test was used to determine whether data obtained was parametric or non-parametrically distributed. A Kruskal-Wallis and post-hoc Mann-Whitney-*U* test was used to determine significant differences between experimental groups where *p* values < 0.05 were considered significant. Means ± standard error is presented.

## Results

### Optimal methylene blue concentration

**Fig. 1:**
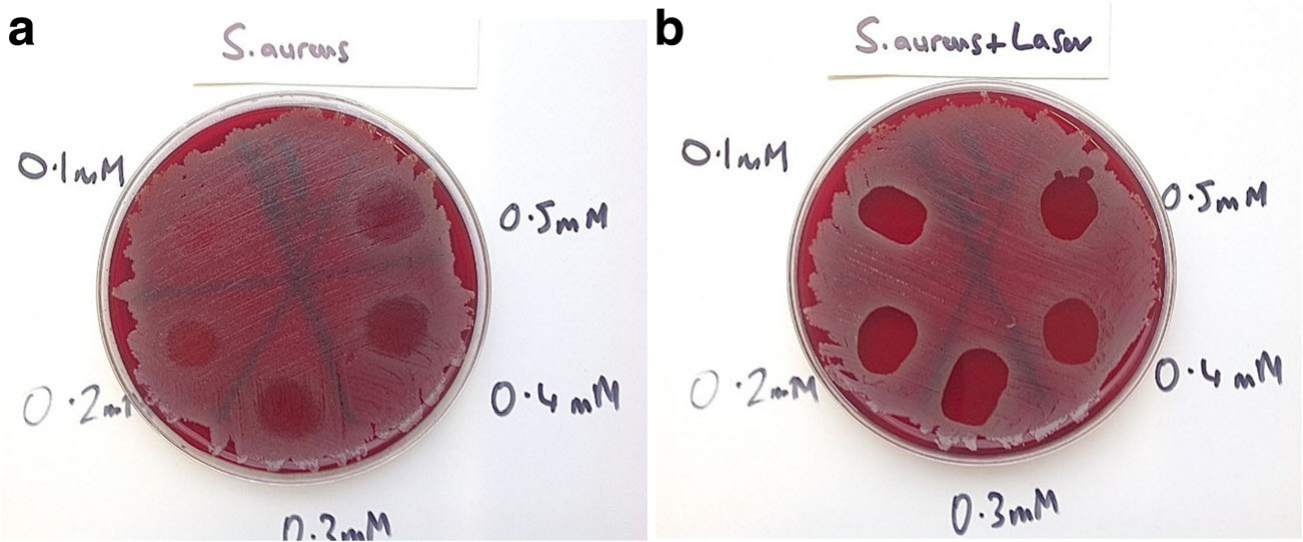
**a**. Photosensitizer MB alone has had a limited bactericidal effect on MSSA. The higher the concentration of MB, the greater the number o bacteria killed. However, there are still significant CFUs remaining even at the highest concentration (0.5 mM). **b**. Photosensitizer MB and laser have completely eradicated all MSSA CFUs at all concentrations of MB (0.1-0.5 mM)

**Fig. 2:**
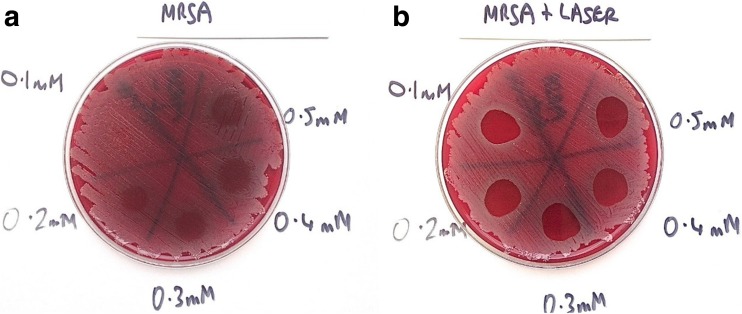
**a** Photosensitiser MB alone of a concentration over 0.2 mM has had a limited bactericidal effect on MRSA. The higher the concentration of MB, the greater the number of bacteria killed but there are still significant CFUs remaining even at the highest concentration (0.5 mM). **b** Photosensitiser MB with laser has completely eradicated all MRSA CFUs with all concentrations of MB (0.1–0.5 mM)

**Fig. 3:**
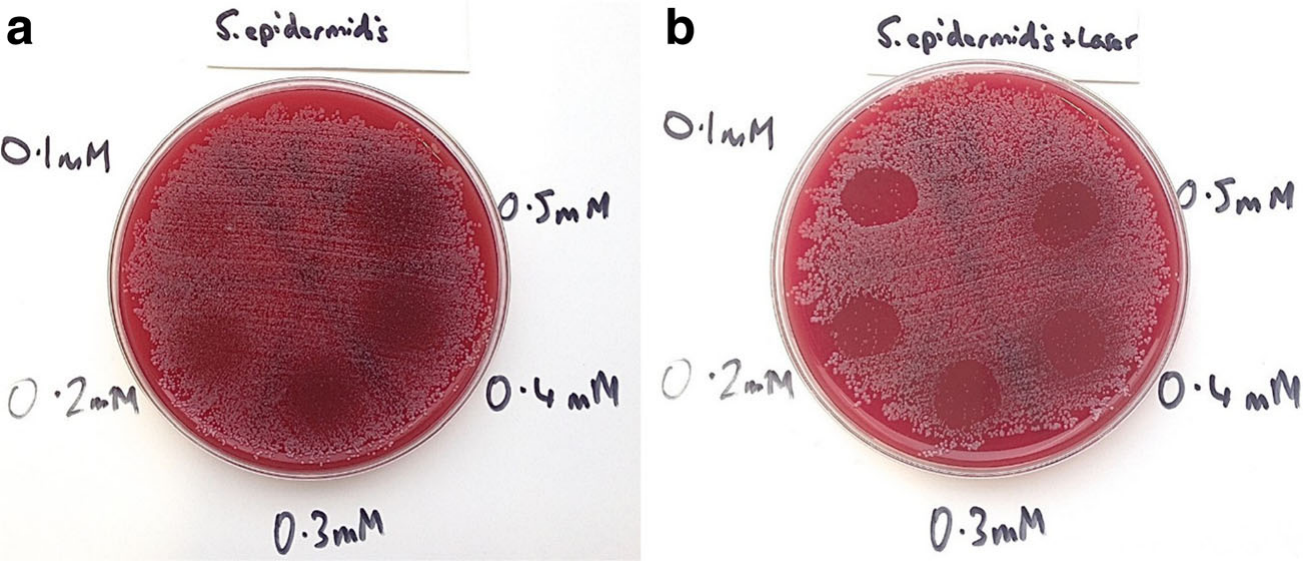
**a** Photosensitiser MB alone of all concentrations has had a limited bactericidal effect on *S. epidermidis* but its effect is not as great as it is for *S. aureus* as there are CFUs scattered throughout the droplet. The higher the concentration of MB, the greater the number of bacteria killed but there are still significant CFUs remaining even at the highest concentration (0.5 mM). **b** Photosensitiser MB with laser has been more effective than MB alone and has killed large numbers of *S. epidermidis* with all concentrations of MB (0.1–0.5 mM). In the higher concentrations of MB (0.4 and 0.5 mM) there is also evidence of bacterial clearance in the margins surrounding the droplet. However, none of the concentrations eradicated all bacteria and there are small numbers of CFUs even with 0.5 mM MB and laser

**Fig. 4:**
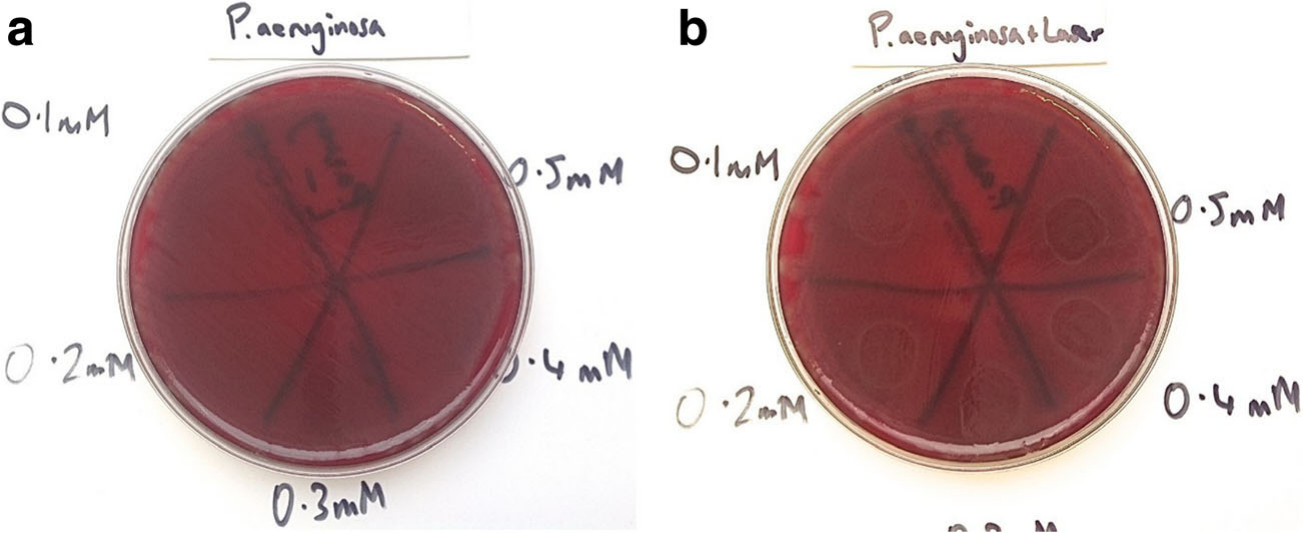
**a** Photosensitiser MB alone has had no bactericidal effect against *P. aeruginosa* at the lower concentrations (0.1 and 0.2 mM). At the higher concentrations (0.3–0.5 mM), there have been a very small amount of bacteria killed with the amount increasing slightly as the concentration of MB strengthens. However, even at 0.5 mM there are large numbers of bacteria still present and the effects of MB alone are negligible. **b** Photosensitiser MB with laser has been more effective than MB alone and some *P. aeruginosa* bacteria have been killed with all concentrations of MB (0.1–0.5 mM). The greater the concentration, the greater the bactericidal effect. However, none of the concentrations eradicated all bacteria and there are widespread CFUs even with 0.5 mM MB and laser

**Fig. 5:**
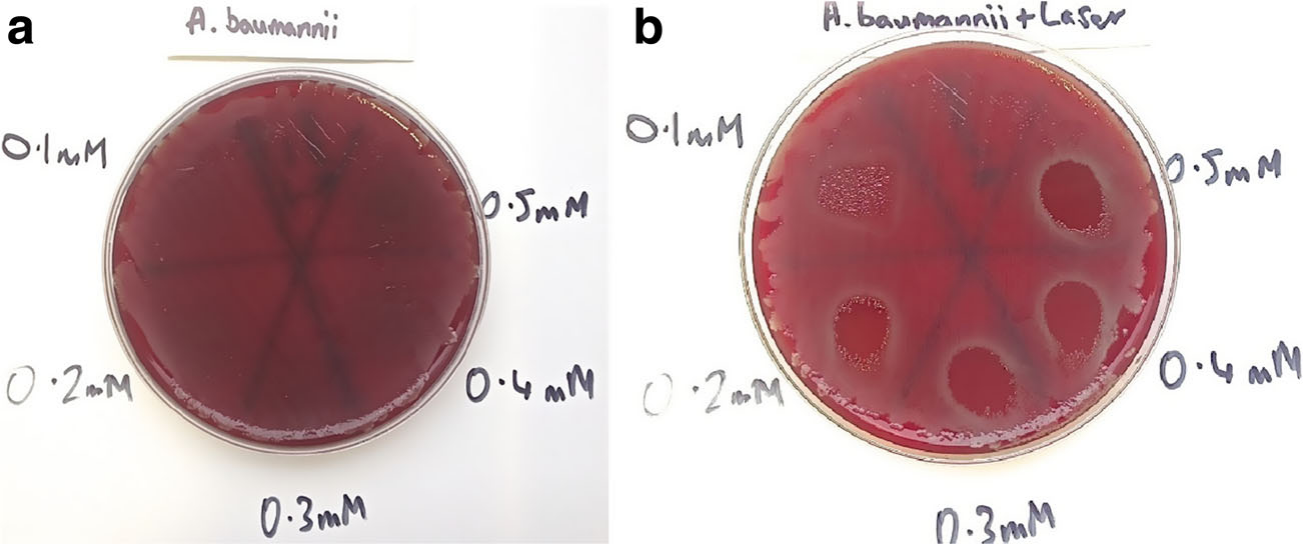
**a** Photosensitiser MB alone has had no bactericidal effect against *A. baumannii* at any concentration of MB. **b** Bacteria have been killed by photosensitiser MB with laser at all concentrations of MB. The greater the concentration the more bacteria were killed. With concentrations of 0.3 mM MB and greater the bacteria were almost eradicated but even at the highest concentration (0.5 mM) there were small numbers of CFUs in the periphery of the droplet

Results showed that in plates treated with MB alone, all Staphylococci were eradicated (MSSA, MRSA and *S. epidermidis*), but the bactericidal effect was limited. For MSSA and MRSA, the greater the concentration of MB used, the greater the bactericidal effect. However, when *P. aeruginosa* and *A. baumannii*, with MB alone were investigated, no bactericidal effect at any concentration was seen. The effect of PDT (MB+L+) is shown in Figs. 1, 2, 3, 4 and 5. When agar plates of MSSA (Fig. 1) and MRSA (Fig. 2) were exposed to PDT, a complete eradication of bacteria within the droplet area was seen. A significant bactericidal effect was also measured with *S. epidermidis* (Fig. 3), but a few resistant colonies remained following treatment in all of the MB concentrations investigated. The effect of PDT on *P. aeruginosa* was very limited with clusters of bacterial colonies still present at all MB concentrations (Fig. 4). The bactericidal effect of PDT on *A. baumannii* (Fig. 5) showed complete clearance of bacteria within the droplet area at the higher concentrations of MB (0.3, 0.4 and 0.5 mM) but little impact on colonies number at the lower concentration (0.1 mM) was seen.

### Optimal laser dose

**Fig. 6:**
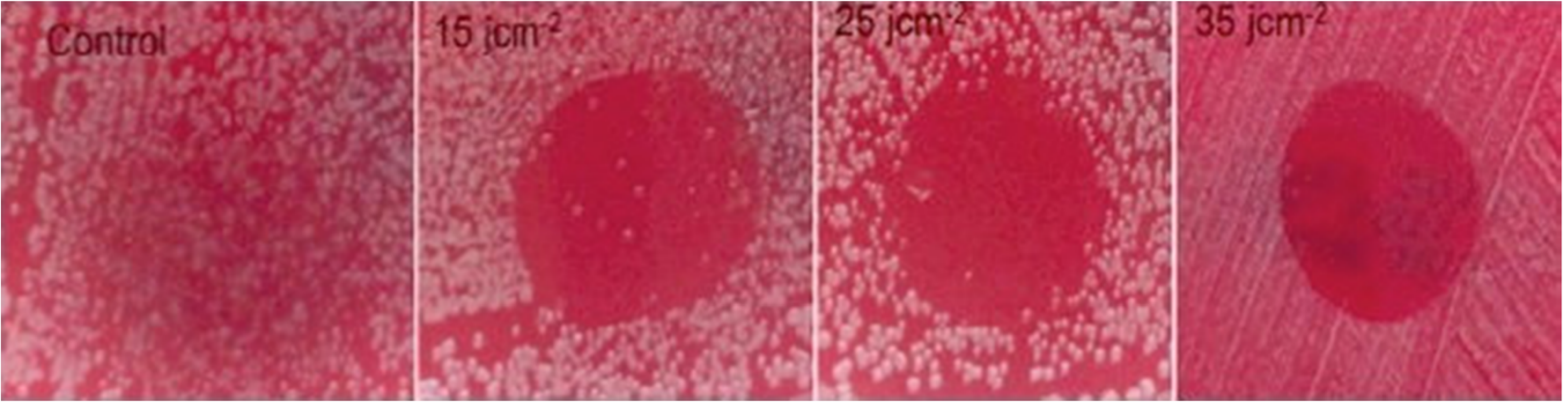
The effect of increasing laser power with 0.3 mM methylene blue on *S. epidermidis* colonies grown on Columbia agar +5% horse blood (power 0, 15, 25 and 35 J/cm^2^)

Results showed that the higher the dose of laser light used, the more bacteria were eradicated by PDT. Control groups where no laser applied, had no effect on bacteria and a laser dose of 15 and 30 J/cm^2^ was effective but a few colonies were observed. The lowest laser dose to eradicate all bacteria was 35 J/cm^2^ (Fig. 6).

### Effect of PDT on planktonic culture

#### Methicillin-resistant *Staphylococcus aureus* (MRSA)

**Fig. 7:**
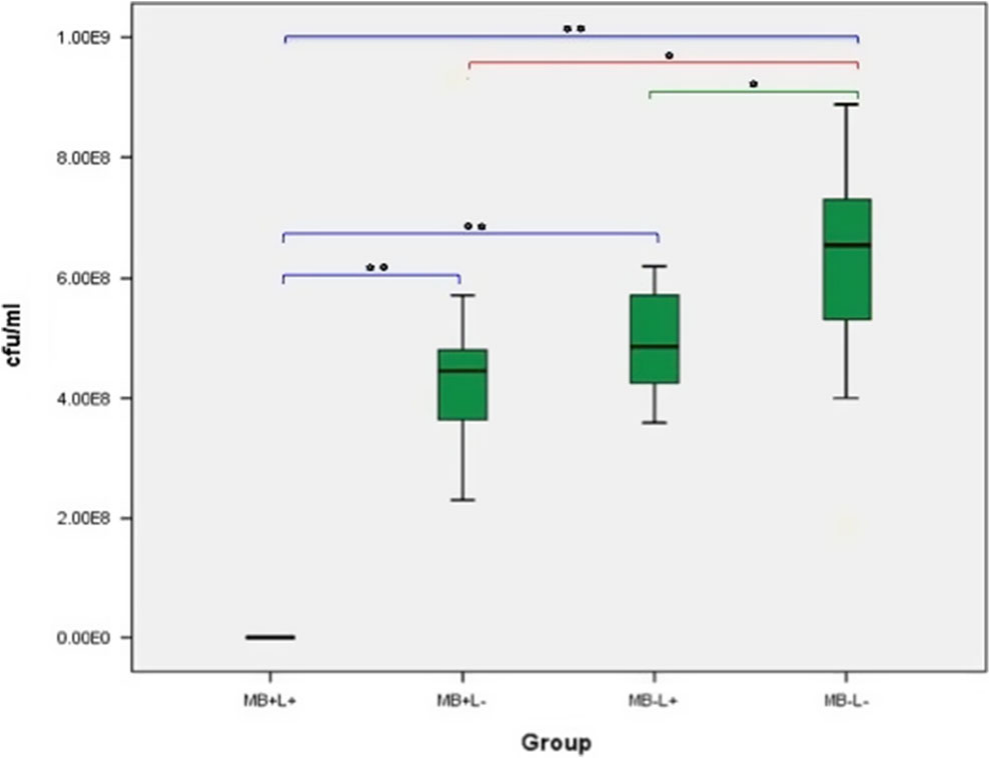
Box plot representing the amount of planktonic MRSA (CFU/ml) remaining within the culture following treatment with PDT (MB+L+), photosensitiser alone (MB+L−), laser alone (MB−L+) and a control (MB−L−). *N* = 12/group. ** *p* < 0.001 * *p* ≤ 0.05

The effect of laser alone (MB−L+), MB alone (MB+L−) or the combination of both (PDT: MB+L+) on MRSA planktonic culture growth is shown in Fig. 7. A significant reduction in MRSA was seen in all three treatment groups when compared to the control group. None of the treatment groups or the control completely eradicated MRSA growth in any samples but PDT significantly reduced bacterial growth when compared either to laser alone (*p* < 0.001) and MB alone (*p* < 0.001) (23,066.67 ± 4379.78 CFU/ml). No significant bacterial reduction was found when the MB alone and laser alone groups were compared.

#### *Staphylococcus epidermidis*

**Fig. 8:**
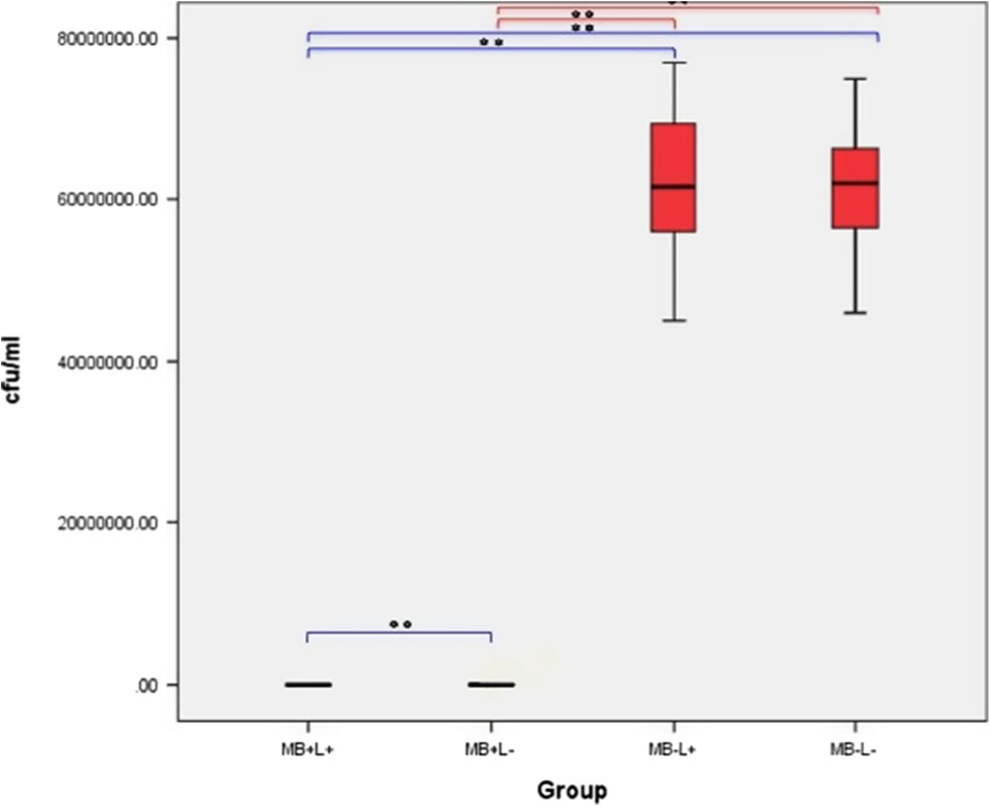
Box plot representing the amount of planktonic *S. epidermidis* (CFU/ml) remaining within the culture following treatment with PDT (MB+L+), photosensitiser alone (MB+L−), laser alone (MB−L+) and a control (MB−L−). *N* = 12/group. **p < 0.001 *p ≤ 0.05

The effect of each of the four treatments on *S. epidermidis* planktonic culture growth is shown in Fig. 8. A significant reduction in *S. epidermidis* growth in both the PDT and MB alone groups was found when compared to control (*p* < 0.001). No significant difference was found when the laser alone and control groups were compared. PDT treatment completely eradicated *S. epidermidis* in all samples. Treatment with MB alone (MB+L−) and treatment with laser alone (MB−L+) did not result in a complete eradication of *S. epidermidis* in any of the 12 samples but MB alone was associated with significantly lower bacterial growth than the control (*p* < 0.001). When the different treatments were compared, MB alone resulted in a significantly greater *S. epidermidis* reduction than laser alone (*p* < 0.001). PDT had a significant bactericidal effect on planktonic culture when compared either to laser alone (*p* < 0.001) or to MB alone (*p* < 0.001).

### Effect of PDT on biofilm (polished titanium alloy discs)

#### Methicillin-sensitive *Staphylococcus aureus* (MSSA) on polished titanium alloy discs

**Fig. 9:**
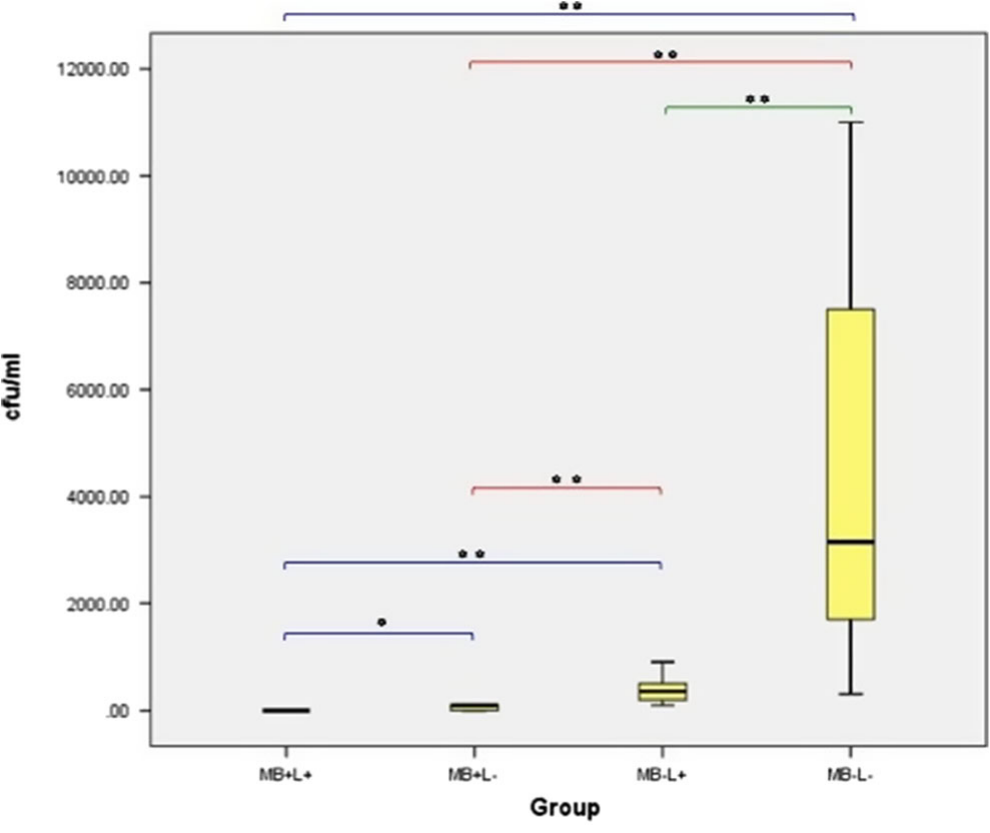
Box plot representing the amount of MSSA (CFU/ml) remaining on the polished titanium disc following treatment with PDT (MB+L+), photosensitiser alone (MB+L−), laser alone (MB−L+) and a control (MB−L−). *N* = 12/group. **p < 0.001 *p ≤ 0.05

The effect of the treatment regimes on MSSA biofilm growth on polished titanium alloy discs is shown in Fig. 9. A significant reduction in MSSA biofilm growth was seen in all three treatment groups when compared to the control group (*p* < 0.001). Treatment with PDT (MB+L+) completely eradicated MSSA in all samples. Treatment with MB alone (MB+L−) also eradicated MSSA in 5 of the 12 samples (58.33 ± 14.86 CFU/ml) and treatment with laser alone (MB−L+) did not result in complete eradication of MSSA biofilm in any sample, but was associated with significantly less CFU/ml bacterial growth than in the control (383.33 ± 70.53 CFU/ml). When the different treatments on biofilm growth were compared, MB alone resulted in a significantly greater MSSA reduction than laser alone (*p* < 0.001). PDT significantly reduced biofilm when compared to both laser alone (*p* < 0.001) and MB alone (*p* = 0.002).

#### Methicillin-resistant *Staphylococcus aureus* (MRSA) biofilm on polished titanium alloy discs

**Fig. 10:**
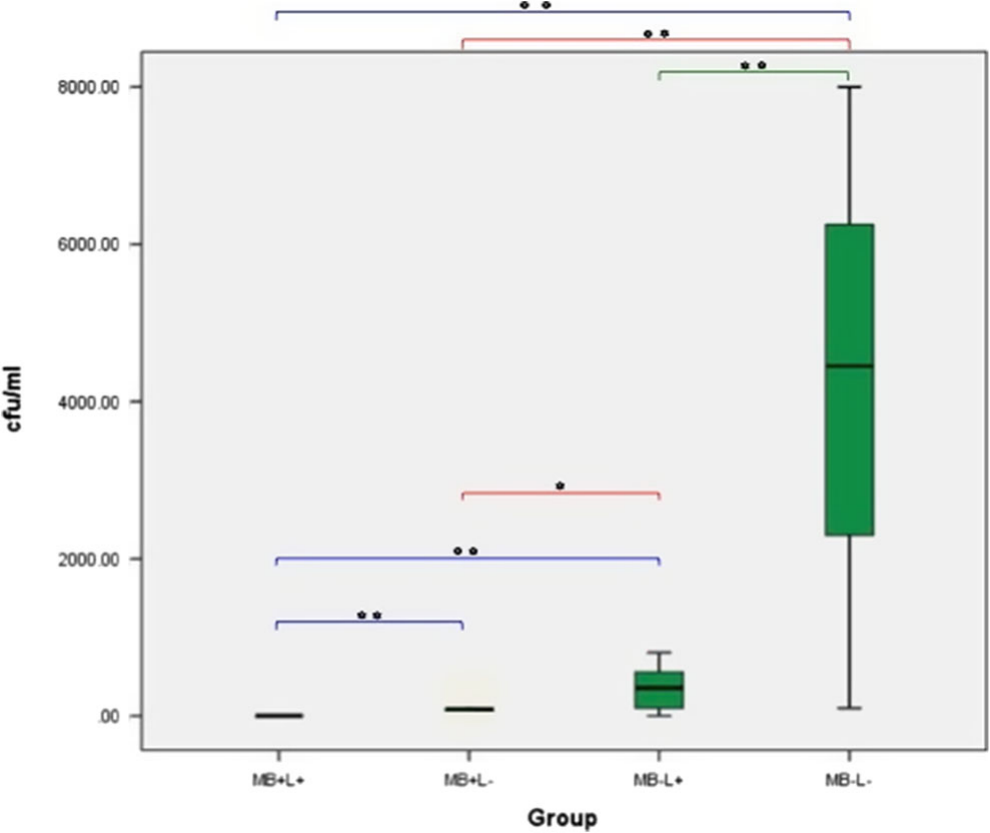
Box plot representing the amount of MRSA (CFU/ml) remaining on the polished titanium disc following treatment with PDT (MB+L+), photosensitiser alone (MB+L−), laser alone (MB−L+) and a control (MB−L−). *N* = 12/group. **p < 0.001 *p ≤ 0.05

The effect of the treatment regimes on MRSA biofilm when grown on polished discs is shown in Fig. 10. A significant reduction in MRSA biofilm was seen in all three treatment groups when compared to the control (*p* ≤ 0.001). Treatment with PDT completely eradicated MRSA in all samples and treatment with MB alone eradicated MRSA in 3 of the 12 samples (91.67 ± 19.30 CFU/ml). Treatment with laser alone eradicated MRSA in just 1 of the 12 samples but was associated with significantly lower biofilm than control samples (341.67 ± 72.26 CFU/ml). When the different treatment groups and the effect on biofilm growth were compared, MB alone resulted in a significantly greater MRSA biofilm reduction than laser alone (*p* = 0.008). PDT significantly reduced biofilm when results were compared both to laser alone (*p* < 0.001) and MB alone (*p* < 0.001).

#### *S. epidermidis* biofilm on polished titanium alloy discs

**Fig. 11:**
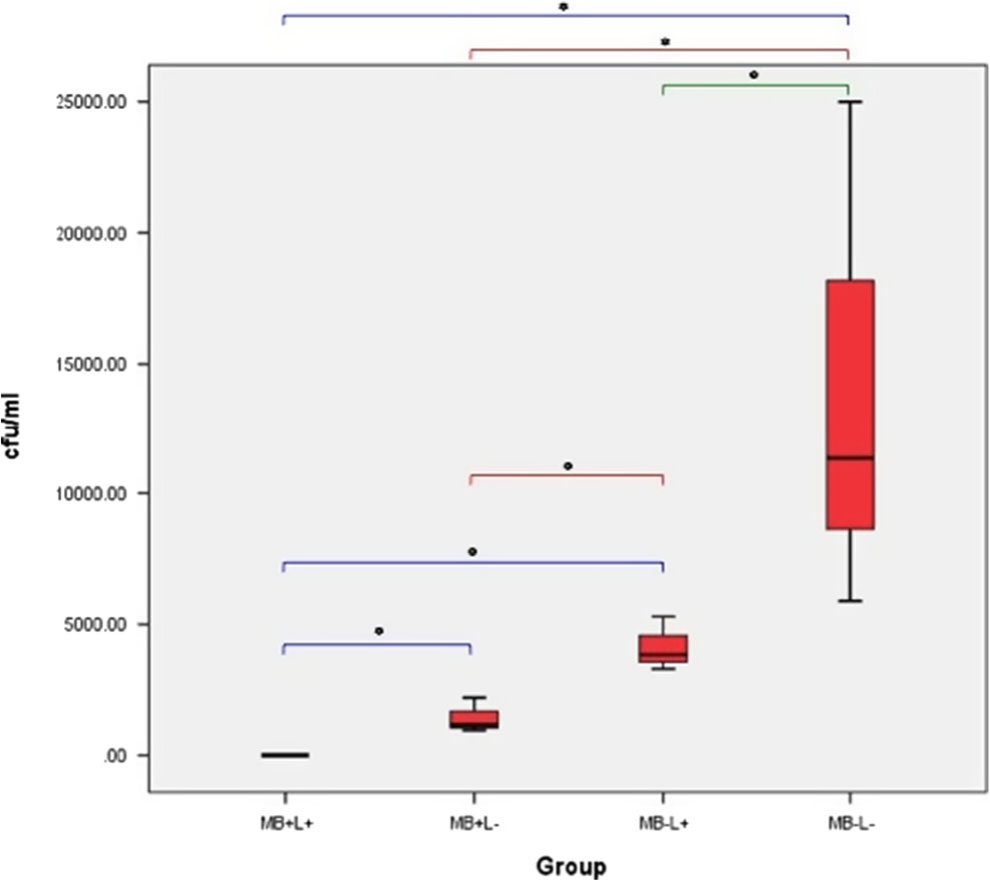
Box plot representing the amount of *S. epidermidis* (CFU/ml) remaining on the polished titanium disc following treatment with PDT (MB+L+), photosensitiser alone (MB+L−), laser alone (MB-L+) and a control (MB−L−). *N* = 3/group. **p < 0.001 *p ≤ 0.05

The effect of treatment regimes on *S. epidermidis* biofilm on polished titanium alloy discs is shown in Fig. 11. There was a significant reduction in *S. epidermidis* biofilm in all three treatment groups when compared to the control group (PDT, *p* = 0.037; MB+L−, *p* = 0.05; and MB−L+, *p* = 0.05). PDT treatment completely eradicated *S. epidermidis* in all samples. Treatment with MB alone and treatment with laser alone did not result in complete eradication of *S. epidermidis* biofilm in any of the 6 samples but was associated with significantly lower biofilm than control. Comparing the effect of the different treatments on biofilm growth, MB alone resulted in significantly greater *S. epidermidis* biofilm reduction than laser alone (*p* = 0.05). PDT significantly reduced biofilm when compared either to laser alone (*p* = 0.037) or to MB alone (*p* = 0.037).

#### *Pseudomonas aeruginosa* grown in biofilms on polished titanium alloy discs

**Fig. 12:**
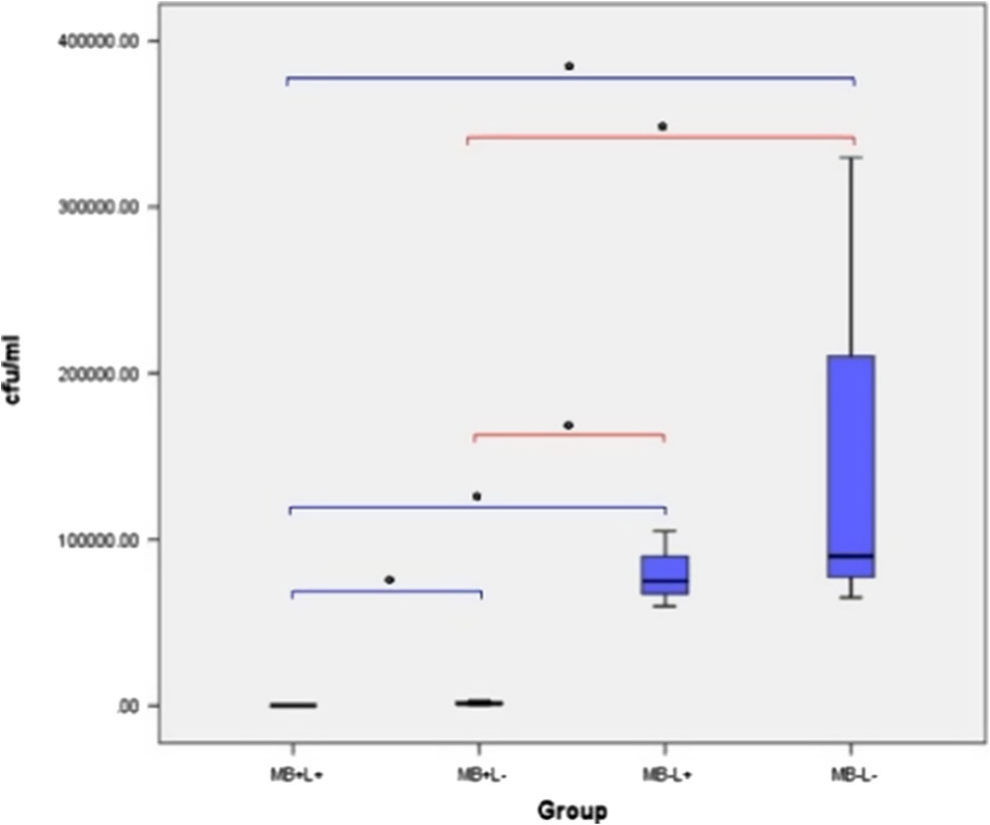
Box plot representing the amount of *P. aeruginosa* (CFU/ml) remaining on the polished titanium disc following treatment with PDT (MB+L+), photosensitiser alone (MB+L−), laser alone (MB−L+) and a control (MB−L−). *N* = 3/group. **p < 0.001 *p ≤ 0.05

The effect of the treatment regimes on *P. aeruginosa* biofilm on polished titanium discs is shown in Fig. 12. Both PDT and MB alone eradicated significantly more bacteria in *P. aeruginosa* biofilm when compared to the control (*p* = 0.037 and *p* = 0.05, respectively). However, samples in the laser alone groups did not show a significant reduction when compared to control. PDT treatment completely eradicated *P. aeruginosa* in all 6 samples and MB alone significantly reduced bacterial growth but did not enable complete eradication of *P. aeruginosa* biofilm in any sample.

#### Effect of PDT on biofilm culture (hydroxyapatite-coated titanium discs)

**Fig. 13:**
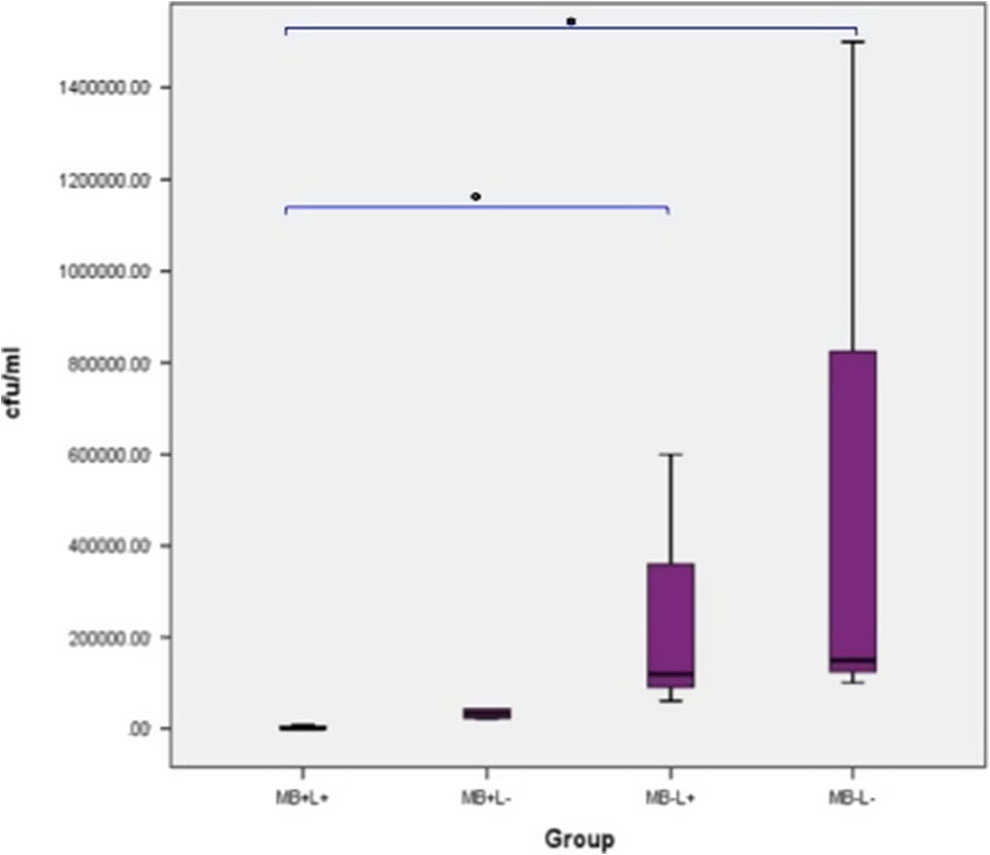
Box plot representing the amount of *P. aeruginosa* (CFU/ml) remaining on the HA-coated titanium disc following treatment with PDT (MB+L+), photosensitiser alone (MB+L−), laser alone (MB−L+) and a control (MB−L−). *N* = 3/group. **p < 0.001 *p ≤ 0.05

The effect of the treatment regimes on *P. aeruginosa* biofilm on HA-coated titanium discs is shown in Fig. 13. A significant reduction in *P. aeruginosa* biofilm was seen when PDT treated discs were compared with the control group (*p* = 0.05). No significant reduction in bacterial recovery was measured in the MB alone or laser alone groups when compared with control samples. PDT treatment eradicated *P. aeruginosa* in only 1 sample (2766.67 ± 2617.30 CFU/ml) and treatment with MB alone and with laser alone did not result in complete eradication of *P. aeruginosa* biofilm in any sample. No significant reduction in bacteria was found when results were compared to the control.

No significant difference in bacterial eradication was seen when MB alone and laser alone groups were compared. PDT significantly reduced biofilm when compared to laser alone (*p* = 0.05) but not MB alone. The effect of the treatment groups on *P. aeruginosa* biofilm when polished discs were compared with the HA-coated discs, are shown in Fig. 13. PDT treated biofilms grown on a polished disc surface showed a significant decrease in *P. aeruginosa* growth when compared with HA-coated surfaces (*p* = 0.037). No significant reduction in bacterial growth was seen when MB alone, laser alone and the control group were compared.

## Discussion

This study aimed to explore the effectiveness of PDT as a means of eradicating strains of bacteria that commonly cause PJIs by forming biofilm on orthopaedic implants. This study clearly demonstrated that PDT is more effective than MB alone for all five bacterial strains investigated. Staphylococci colonies were eradicated even at the lowest concentration of MB (0.1 mM). However, with *P. aeruginosa* and *A. baumannii*, increasing the concentration of MB improved the bactericidal effect, which indicated that different bacteria species and even strains may respond differently to PDT.

Previous studies have had success using lower concentrations of MB. Tanaka et al. demonstrated that the optimal concentration of MB to use against planktonic MRSA in vitro was 0.1 mM [19]. Similarly, a study by Usacheva et al. showed that a concentration of between 0.025 and 0.044 mM of MB was most effective at eradicating Gram-positive bacteria, whilst concentrations between 0.015 and 0.250 μM were optimal for Gram-negative bacteria [24]. In this study, we found an optimal concentration of 0.3 mM MB. Future studies are needed to quantitatively determine the optimal concentration of photosensitizer required for use during in vivo applications.

Increasing the laser power increased the bactericidal effect of PDT with a dose of 35 J/cm^2^ and above, successfully eradicating all colonies of *S. epidermidis*. Other studies have also shown that increasing the intensity of light, whilst keeping the photosensitizer concentration constant, results in greater bacterial destruction [24]. For this reason, a 35-J/cm^2^ laser dose was used in our subsequent studies. Other studies have shown that increasing the time of PDT light exposure significantly elevated the bactericidal effect [25].

The effective antimicrobial effect of PDT on planktonic culture was demonstrated through its ability to significantly reduce MRSA numbers (CFU/ml) from millions to thousands and to completely eradicate *S. epidermidis*. Similar results have also been demonstrated in the destruction of *S. aureus* culture using PDT [26].

In this study, the use of PDT significantly reduced bacterial numbers compared to treatment with photosensitizer alone, laser alone and the control (no treatment). This is in keeping with previous studies that investigated the ability of PDT to eradicate bacterial biofilm growth in dental plaques, on oral implants [26, 27] and within chronically infected wounds [28, 29]. Although the bactericidal effect of PDT had a significant effect on bacteria in both planktonic culture and within biofilm, it appeared to be more effective against biofilms which given the resistance of bacteria within biofilms, this result was not expected. Complete eradication of bacteria within a biofilm is important because microbial biofilms can quickly re-form unless all bacteria are killed. The success of PDT demonstrated by these results is also supported by Biel et al. who tested PDT on polymicrobial biofilms (MRSA and *P. aeruginosa*) within endotracheal tubes using MB and a 664-nm light via a small optical fiber. In this study, PDT was shown to reduce the number of bacteria within the biofilm by over 99.9% after a single treatment [30].

PDT was less effective when eradicating bacterial biofilms grown on HA-coated discs. A reason for this may be due to the increased surface area present on HA-coated surfaces. Several studies have shown that implant surface characteristics effect the bacterial growth rate and survival [31] and a study using electron microscopy demonstrated high bacteria adherence to HA-coated surfaces [32, 33]. Furthermore, PDT might not be as successful because it relies on light reaching the affected, photosensitised area to form singlet oxygen. On a roughened surface with many pores, there may be some areas where light is unable to reach.

Our study demonstrated that PDT was effective in killing Gram-positive and Gram-negative bacteria that cause PJI when in biofilms. It is speed of action and lack of bacterial resistance may make PDT a beneficial and safe treatment for treating infected implants during revision procedures. There is an increased risk of reinfection after revision procedures, PDT may be able to reduce the bacterial burden further during these procedures. When the implant is changed, PDT could be used to disinfect surrounding soft tissues and bone. The data acquired in this study should be used to investigate and eradicate PJI in in vivo models before being used in a human clinical trial.
